# The Development and Validation of a Disordered Eating Screening Tool for Current and Former Athletes: The Athletic Disordered Eating (ADE) Screening Tool

**DOI:** 10.3390/nu16162758

**Published:** 2024-08-19

**Authors:** Georgina L. Buckley, Annie-Claude M. Lassemillante, Matthew B. Cooke, Regina Belski

**Affiliations:** 1 Body Justice, Victoria St., Fitzroy, VIC 3065, Australia; 2 Department of Nursing and Allied Health, Swinburne University of Technology, John St., Hawthorn, VIC 3122, Australia; 3 Sport, Performance and Nutrition Research Group, School of Allied Health, Human Services and Sport, La Trobe University, Plenty Rd. & Kingsbury Dr., Bundoora, VIC 3086, Australiamatt.cooke@latrobe.edu.au (M.B.C.)

**Keywords:** disordered eating, screening tool, Rasch analysis, validation methods, Item Response Theory, scale development, eating disorders, athlete, body image

## Abstract

Background: Current and former athletes are one of the most at-risk population groups for disordered eating (DE), impacting their dietary practices, body composition, performance and health during and following their athletic careers. Few comprehensive DE screening tools exist for this group. To help address this, the current study utilised a mixed-methods approach of Classic Test Theory (CTT) and Item Response Theory (IRT) to develop and validate a DE screening tool suitable for current and former athletes. Methods: Novel scale development methodologies were used to develop and assess the validity (content, face, cross-cultural, construct), test-retest reliability, internal consistency reliability, factor analysis and Rasch analysis of a new DE scale. Results: A new validated Athletic Disordered Eating (ADE) screening tool was created, with 17 items and four subscales (food control, bingeing, body control, body discontent), with an internal consistency reliability of 0.91, excellent content and construct validity, an Intraclass Correlation Coefficient of 0.97 and excellent Rasch model fit. Conclusions: The ADE screening tool has been dually developed for research purposes and as a clinically applicable screening tool to detect DE in current and former athletes and is suitable for a global use across sporting categories, diverse genders and levels of competition.

## 1. Introduction

Athletic populations are one of the most at-risk population groups for eating disorder (ED) development, yet there are significant research gaps in the conceptualisation, understanding and screening of EDs amongst these groups [1,2,3]. In addition, there are subcultural layers that also exist within athletic populations that can further enhance the risk, including athletes who engage in leanness- or thinness-promoting sports, individuals within the early stages of retirement or transitioning out of their sport (whether that be for illness, injury, occupational or parental needs) and athletes who feel a sense of pressure to change their body composition for their sport [3,4,5]. Eating disorders are defined by the Diagnostic and Statistical Manual (DSM) as “a persistent disturbance of eating or eating-related behaviour that results in the altered consumption or absorption of food and that significantly impairs physical health or psychosocial functioning” [6]. Disordered eating (DE) on the other hand is a subclinical form of an ED that precedes or proceeds ED development. Eating practices, that would be considered disordered, are commonly reported by athletes, and DE is often a precursor for the development and diagnosis of EDs. The ability to screen athletes for DE with the view to be able to offer early intervention to those at highest risk is a critical step in prevention of ED development. To date, there is a limited range of screening tools that have been developed for athletes in the ED/DE field, especially those that consider and include male athletes, disabled athletes, retiring athletes and gender diverse athletes [7,8].

Numerous methodologies exist in scale development that have been used to capture the latent variable of an ED [7]. The Eating Attitudes Test (EAT-26) [9], Eating Disorder Examination Questionnaire (EDE-Q) [10] and Eating Disorder Inventory-3 (EDI-3) [11] are the main ED scales that have been relatively well used and tested in athletes, whilst the Athletic Milieu Direct Questionnaire (AMDQ) [12], Female Athlete Screening Tool (FAST) [13], Brief Eating Disorder in Athletes Questionnaire (BEDA-Q) [14] and Eating Disorders Screen for Athletes (EDSA) [15] have been developed specifically with athletes in mind. The majority of scales have focused on EDs and not DE. All these scales have used Classic Test Theory (CTT) in their development, whilst none in the ED field have used Item Response Theory (IRT) and rarely have qualitative or mixed-methods been applied to inform the conceptual development of their scale.

Both CTT and IRT are theories that govern methods and statistics related to validity, reliability and psychometric properties [16,17]. CTT is based on the theoretical assumptions of parallel tests, suggesting that each item is equally representative of the latent variable; whereas, IRT is a model that suggests that each item can represent differing amounts of the latent variable [16,18,19]. IRT has emerging psychological applications as it aligns with theory related to items having their own characteristic sensitivity [18]. This is a particularly significant consideration when developing a psychological screening tool where an emphasis on highlighting individuals with differing levels of DE severity is aimed. The IRT model allows for individuals across a spectrum of severity to be detected as opposed to the CTT model, which characterises people with a high severity well and people with subclinical levels poorly or not at all [16]. Importantly, in settings that require quick methods of screening, IRT model encourages the least number of items, but still captures the broadest range of construct domains. Currently, there are no published scales that exist in the ED/DE literature that have utilised IRT [7]. IRT offers an opportunity for development in the ED/DE field as it can be easily used with diverse and under-represented population groups.

Therefore, the aims of this study are to, firstly, utilise novel IRT methodology in combination with CTT in DE scale development and, secondly, to validate its application within a sample of mixed gender, age, sport category and athletic status. The findings of this study will demonstrate how mixed methodology can be utilised alongside IRT to develop the latent variable understanding of DE in under-represented research population groups such as current and former athletes.

## 2. Methods

### 2.1. Methodology and Defining Disordered Eating

**Figure 1:**
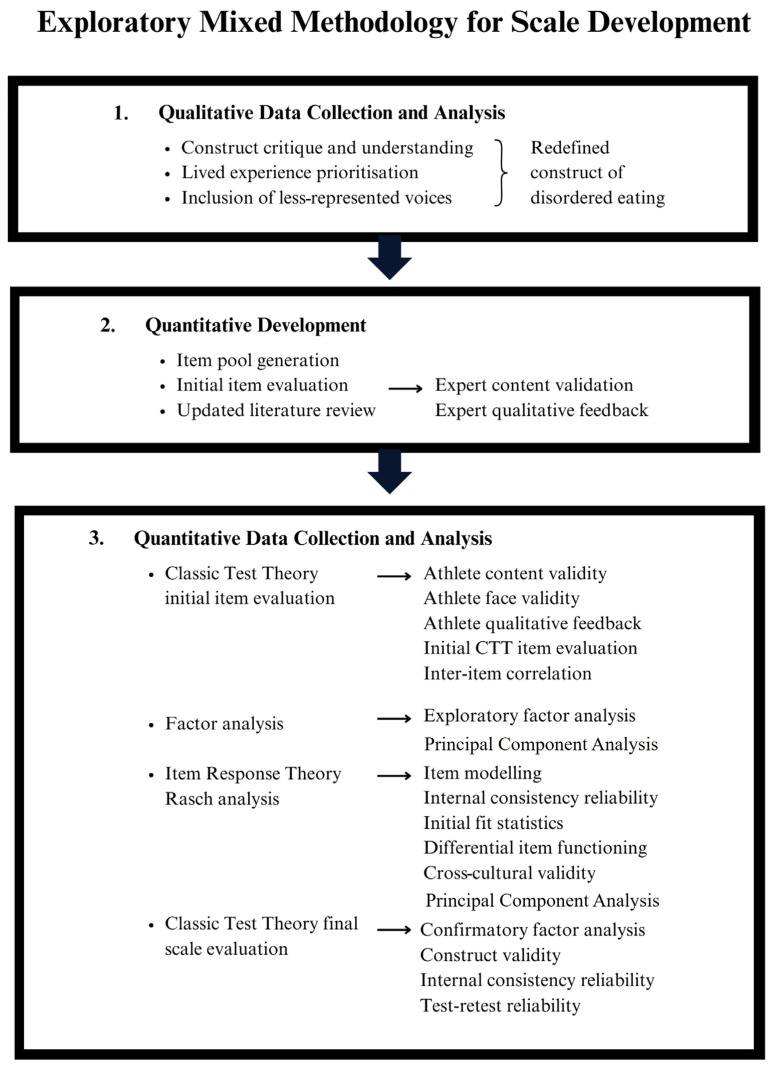
The overall exploratory sequential mixed-methods design project to reconceptualise disordered eating for the development and evaluation of a screening tool.

**Figure 2:**
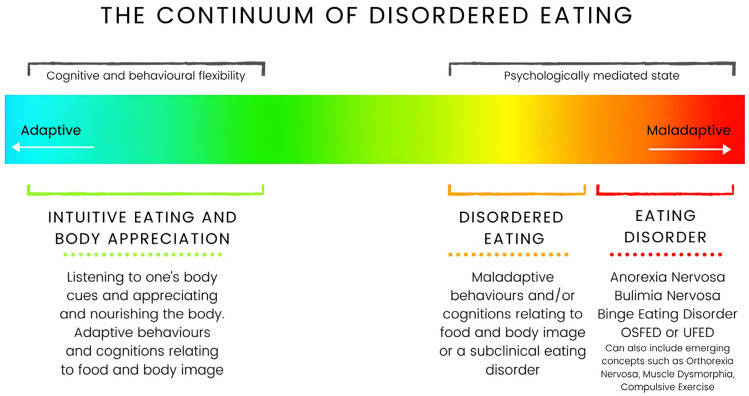
Continuum of disordered eating—from adaptive intuitive eating and body appreciation to maladaptive states of disordered eating and clinical eating disorders [7].

To develop a novel DE screening tool, an exploratory sequential mixed methodology was used for scale development. Three stages were combined, (1) qualitative data collection and analysis, (2) quantitative development and (3) quantitative data collection and analysis, to develop the final scale and combined interpretations (Figure 1). Stage 1 has been reported elsewhere [7], and stages 2 and 3 are presented in this paper. We emphasised the importance of mixed methodology and qualitative methods to prioritise lived experience and to support individuals who have marginalised intersectional identities or those who have limited empirical evidence [20,21,22,23]. In stage 1, a particular emphasis on challenging the dated constructs of EDs and DE was undertaken through a thorough modern literature review and qualitative exploration [7]. We challenged the historical constructs of EDs that emphasise the femme thin ideal, restrictive low-weight anorexia nervosa and cis-female presentations [11,24,25,26] from the bottom up to cater for a broader, more inclusive population definition [7]. Importantly, the first stage culminated in a new construct definition of DE alongside 10 domains, which was used to inform the quantitative development and analysis of stages 2 and 3. The new construct definition states ‘Disordered eating is a state either proceeding or preceding a clinical eating disorder that is cognitively and/or behaviourally mediated and is contextual to an individuals’ sociocultural positioning [7]. The continuum of DE is presented in Figure 2. When compared to EDs, DE is more transient and has less psychological mediation. DE can be experienced by cognitions with the absence of behaviours, as it can be experienced as a subjective perception without the need to explicitly act. When considering the specific state of an individual experiencing DE, the sociocultural positioning and sociocultural influences that may contribute to DE (e.g., objectification, weight stigma, etc.) must always be considered. DE can be made up of any combination of the following 10 domains: dietary control, food obsession/preoccupation, bingeing, restriction, food rules, body control, body discontent, exercise energy control, fat phobia and body idealisation. 

### 2.2. Scale Development

A comprehensive methodological collaboration between CTT and IRT was chosen for the quantitative development and validation of the Athletic Disordered Eating (ADE) screening tool. Classic Test Theory is the most common methodology involved in the development and validation of scales [16]. Many scales that exist in the ED literature choose only a handful of statistical analyses or focus on the most theoretically robust statistics (factor analysis, construct validity, internal consistency reliability) [7,27,28]. We aimed to incorporate as many of the available CTT statistical analyses as possible to ensure that the scale was assessed broadly, could be compared to any currently available ED scales and that future empirical assessment had accessible statistics as a benchmark [27,29]. We utilised content analysis, content validity, exploratory factor analysis, criterion-related validity, construct validity, internal consistency reliability, test-retest reliability and measurement error in alignment with the Consensus-based Standards for the selection of Health Measurement Instruments (COSMIN) quality assessment checklist to ensure a robust CTT methodology was undertaken [27,29]. We recommend that the COSMIN tool is used for critical scale appraisal or future scale development, especially in the field of EDs.

### 2.3. Participant Recruitment

Four participant groups were recruited for the following statistical analyses (Table 1). Participant numbers were recruited in relation to the number of proposed items for the final scale in accordance with the COSMIN Study Design Checklist (Appendix A) [16,27,30,31].

### 2.4. Participant Demographics

Group 1—Expert validation evaluation occurred in June 2019 and had representation from 3 different countries: Australia (*n* = 7), the USA (*n* = 3) and the UK (*n* = 1) and included *n* = 8 females and *n* = 3 males. 

Group 2—The pilot study of current and former athletes over the age of 18 was conducted in April of 2020. A total of *n* = 68 participants started the survey, with 17 being excluded for not completing the questionnaire. The sample included *n* = 7 males, *n* = 42 females and *n* = 2 gender diverse individuals from Australia (*n* = 25), the USA (*n* = 17), the UK (*n* = 4), Sweden (*n* = 1), Ireland (*n* = 1), Czech Republic (*n* = 1), Canada (*n* = 1) and Albania (*n* = 1). There was *n* = 23 current athletes and *n* = 28 former athletes from antigravitational (*n* = 2), ball (*n* = 9), endurance (*n* = 27), power (*n* = 5), weight class (*n* = 4) and other (*n* = 4) categorised sports. Only *n* = 2 participants identified as current or former para-athletes.

Group 3—The development sample recruitment of current and former athletes over age of 18 was conducted from April to September of 2020. A total of *n* = 1464 participants began the survey, with only *n* = 851 completing sufficient detail to be included in the analysis. Length of survey was the main reason for survey non-completion. Participants completing at least one scale of the battery (ADE item pool, EAT-26, BEDA-Q, IES-2 or BAS-2) were included. The final analysis sample included *n* = 239 current female athletes, *n* = 238 former female athletes, *n* = 216 current male athletes and *n* = 150 former male athletes. In addition to these male and female categories, there were *n* = 5 non-binary, *n* = 1 transgender and *n* = 2 individuals who did not identify with the gender options present. Individuals outside of the male and female dichotomy were included when the whole population was analysed but excluded when looking specifically at the 4 population groups. The participants were aged between 18 and 70 years (M = 25.2 years, SD = 8.0). Amongst the former athletes (*n* = 388), 1.3% (*n* = 11) retired within the last 3 months, 2.2% (*n* = 19) between 3 and 6 months, 4.1% (*n* = 35) 6 and 12 months, 9.8% (*n* = 83) 1 and 2 years, 13.3% (*n* = 113) 2 and 5 years, 11.0% (*n* = 94) 5 and 10 years and 4.5% (*n* = 39) retired greater than 10 years. There was *n* = 15 para-athletes included in the sample. Further demographic data are presented in Table 2.

Group 4—A sample of *n* = 125 participants completed the item response pool again from 1 to 3 weeks after the first time of completion. These participants were recruited from the previous development sample and sent an email 7 days after their first completion of the development scale and a reminder email at 14 days. Participants were excluded (*n* = 48) if they did not complete all responses to the item pool. Of those that completed the test-retest assessments, 31.2% (*n* = 39) were males, 67.2% (*n* = 84) were females, 1.6% (*n* = 2) were non-binary, 55.2% (*n* = 69) were current athletes and 44.8% (*n* = 56) were former athletes. 

### 2.5. Item Pool Generation

Items were developed deductively from the previous literature and existing ED scales and inductively from the reconceptualised definition of DE [7]. One hundred and six initial items were developed as per DeVellis’ (2012) [16] recommendations to capture aspects broadly related to DE and the relevant 10 domains. A test plan was developed with the items to ensure that there was a broad item representation across the defined domains. Items were discarded if they were not generalisable for former athletes, para-athletes, gender diverse participants and individuals with varying sociocultural backgrounds. Reverse-scored items were included [16], and temporal considerations were developed to be accurate to 7–21 days [16].

In the development of this DE tool, consideration was given to differing body ideals to avoid using vocabulary that emphasised a ‘thin ideal’ and to include former athletes in all responses. For example, ‘I feel anxious about my body shape whilst competing’ is an item that may not cater adequately for former athletes. In a DE tool, items that emphasise feeling dissatisfaction about certain body parts (such as legs or arms) may be irrelevant for certain para-athletes or be too specific for people who feel dissatisfied about differing aspects of their body. For example, a male distance runner who experiences DE might experience body dissatisfaction related to their calf muscularity, whereas a female distance runner might experience more body pressures related to their overall physical appearance of their abdomen. 

### 2.6. Classic Test Theory Initial Item Evaluation

An initial evaluation highlighted poorly fitting items for omission. Content validation was assessed amongst experts with items being discarded for having an Item Content Validity Index < 0.78 or poor qualitative feedback [31,32]. Qualitative content analysis (“how relevant is this item?”) and face validity (“is this item well worded?”) were conducted amongst current and former athletes to rank items for consideration with the remaining statistical tests [17,33]. An initial item evaluation (response frequency, mean score, variance, standard deviation, inter-item correlation) was conducted using SPSS v25.0 [34]. Inter-item correlation was assessed with items being discarded for having a high correlation > 0.7, a negative value, low correlation < 0.3 or a poorly corrected item–total correlation [16,17,35]. Items were ranked and flagged for potential future disposal [17] that had a high variance with mean scores falling significantly outside of the centre of the range.

### 2.7. Factor Analysis

Factor analysis was conducted to assess the unidimensionality of the scale [17]. Exploratory factor analysis (EFA) and factor rotation assessed the dimensions (factors) involved in the scale items making up the expected latent variable of DE. Three statistics determined if the items were suitable for factor analysis; they included Kaiser–Meyer–Olkin (KMO) > 0.8, Bartlett’s test of sphericity of *p* = 0.000 and Anti-Image Measure Sample Adequacy > 0.09 [16,36]. Principal Component Analysis (PCA) was used to determine the number of factors amongst the scale [35] through collaborative judgement of Kaiser’s Criterion of eigenvalues, Cattell’s scree plot [37], percentage of variance [38] and Horn’s parallel analysis [39]. Once the factors were decided, EFA rotations determined which of the items load most strongly onto each of the specific factors. Oblimin Factor Rotation was then conducted to assess each remaining item for their specific correlation to the final number of factors; correlations < 0.4 were omitted [17].

### 2.8. Rasch Analysis

RUMM2030 [40] was used to analyse the item modelling and internal consistency reliability through the Person Separation Index (PSI) [17,41]. Initial fit statistics (item–person interaction, item fit and item trait through residuals and chi squares) were analysed each time an item was removed to assess the overall scale improvement to the Rasch model [41]. Item misfit was signified through a poor fit to the item–characteristic curve and was omitted [17]. Items were additionally omitted if they contributed to a worsened item fit (i.e., poor item probability, poor item curvature, poor DIF, poor discrimination fit, local dependence > 0.2, poor individual person fit) [17,41]. Cross-cultural validity was achieved through Multi-Group Confirmatory Factor Analysis as part of the Differential Item Functioning (DIF) assessment [42]. DIF highlighted when there were discrepancies between groups and the model and identified items that were not generalisable across all groups [43]. We used DIF to analyse sex (males vs females) and athletic status (current vs former athletes). Items were discarded if they had a disordered threshold map (the distribution of responses from never to always) [41]. Principal Component Analysis was then used to assess the final scale’s dimensionality with items being discarded if their local dependency was >0.2 [17].

### 2.9. Classic Test Theory Final Scale Evaluation

A final analysis was conducted on the final scale items to determine how the overall scale functioned. Criterion-related validity (predictive validity) was analysed by comparison of the final scale items through Receiver Operator Curves with the EAT-26 [9]. The EAT-26 was chosen for its feasibility and better psychometric properties for athlete populations when compared to the EDE-Q [1,44]. Construct validity was tested against the Intuitive Eating Scale (IES-2), Body Appreciation Scale (BAS-2), BEDA-Q and the EAT-26 [9,14,45,46] to determine positive or negative convergence hypothesis testing. We predicted the BAS-2 and IES-2 would negatively correlate and the EAT-26 and BEDA-Q would positively correlate to the final scale items. Correlation coefficients were interpreted as strong if ≥0.9, fairly strong if between 0.8 and 0.9 and moderate if between 0.6 and 0.8 [16]. Confirmatory Factor Analysis was used to review the factor loading of the final items and to transform these factors into meaningful DE subscales. 

Internal consistency reliability was assessed for each of the 4 subgroups across each of the 4 subscales. Nunnally [31] indicates that a score greater than 0.7 is optimal, with scores >0.9 indicating the scale should be shortened in length [16]. The mean inter-item correlations were additionally calculated to further demonstrate internal consistency. An optimal statistic ranges between 0.2 and 0.4 for scales and subscales within populations [16]. 

Test-retest reliability was undertaken on *n* = 125 participants who completed the ADE screening tool twice with a mean time of 13.4 ± 8.5 days between the first and second completion of the scale. The Intraclass Correlation Coefficient (ICC) was assessed to determine the accuracy of the temporal stability [47]. Measurement error was determined from the test-retest data, and the analysis between the two periods indicated a Standard Error of Measurement (SEM), which can be then utilised to calculate the minimum detectable change (MDC) across the total scale and relevant subscales. The SEM is representative of the margin of error by chance involved in administering the scale at multiple times, and the MDC can indicate when respondent score changes are clinically significant or not [17].

## 3. Results

### 3.1. Iterative Item Response Pool Process

**Figure 3:**
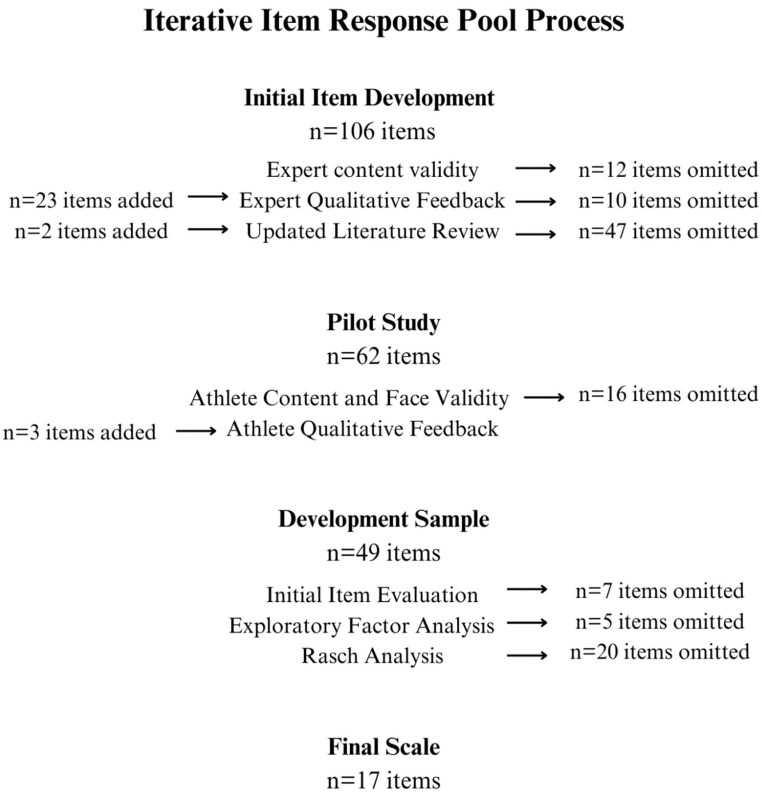
Summary of the iterative item response pool process including omissions and additions.

In summary, a total of *n* = 106 items were initially evaluated, with an additional *n* = 25 items added during the process. A total of *n* = 117 items were omitted systematically through content validity, expert and athlete qualitative feedback, literature revision, face validity, item evaluation, exploratory factor analysis and Rasch analysis, leaving *n* = 17 items for final scale. A summary can be found in Figure 3.

### 3.2. Classic Test Theory Initial Item Evaluation

Expert content validity discarded 12 items for their low Item Content Validity Index (I-CVI), and qualitative expert feedback led to 10 items being omitted and 23 items being added. Following this, a further detailed literature review led to 47 items being discarded and the addition of 2 new items. Content and face validity with current and former athletes led to the omission of 16 items (4 for low I-CVI values, 2 for low face validity and 10 for both low I-CVI and face validity) and an addition of 3 items in relation to the item’s suitability with the four subgroups (current females/males, former males/females). Forty-nine items underwent an assessment through CTT analyses (response frequencies, means, variance, standard deviation and inter-item correlation) with seven being omitted for their poor inter-item correlations or corrected inter-item correlations. Other statistics were used to rank the items and highlight any future potential for discard.

### 3.3. Factor Analysis

We settled on four factors for the final scale through the collaborative judgement of the following statistics. Kaiser’s Criterion of eigenvalues indicated the following factors for each of the subgroups: all participants = 6, current male athletes = 8, former male athletes = 10, current female athletes = 5 and former female athletes = 6. Breaks in Cattell’s scree plot indicated the following factors: all participants = 3, current male athletes = 4, former male athletes = 3, current female athletes = 3 and former female athletes = 3. The percentage of variance indicated that up to 10 factors could be used. Horn’s parallel analysis indicated the optimal factors in the following groups: all participants = 4, current male athletes = 3, former male athletes = 3, current female athletes = 4 and former female athletes = 2. The final eigenvalues utilised with a four-factor scale were 1.62 for all participants (1.67 for current males, 1.67 for former males, 1.70 for current females and 1.59 for former females). This eigenvalue explains an accumulative variance percentage of 56%. Five items were omitted through Oblimin Factor Rotation analyses given their low correlation to one specific factor (<0.4).

### 3.4. Rasch Analysis

Twenty items were omitted through iterative Rasch analysis, and a summary of the model fit statistic for the final seventeen items is indicated in Table 3. All final items indicated a good fit residual to the Rasch model. The Person Separation Index (PSI) indicated internal consistency of the scale and subscales, indicating that it is suitable for four or more population groups with excellent internal consistency validity. It should be noted that the standard deviation of the item fit residual in factors two and three is good but not excellent as they exceed 1.5. In both of these factors, items were carefully balanced to not offset a poor coefficient alpha for the scale nor by losing essential conceptual contributions to the subscale. We omitted 14 items for their poor DIF. No final items had a disordered threshold map or probability curve with disordered threshold either. PCA indicated unidimensionality amongst the four factors, a significant test result was indicated by <5% (Factor 1 = 1.88%, Factor 2 = 1.65%, Factor 3 = 3.93% and Factor 4 = 2.01%).

### 3.5. Classic Test Theory Final Item Evaluation

Criterion-related validity determined the following cut-off scores, as indicated in Table 4. Moderate DE (high sensitivity, low specificity) was indicated by a score of 25 or greater. Spearman correlation coefficients were utilised to determine the construct validity with a moderate positive correlation with the EAT-26 (0.774, *p* = 0.000), a moderate inverse correlation to the IES-2 (−0.732, *p* = 0.000), a moderate inverse correlation to the BAS-2 (−0.666, *p* = 0.000) and a moderate positive correlation to the BEDA-Q (0.657, *p* = 0.000). Confirmatory Factor Analysis determined a Component Correlation Matrix of the four factors (Table 5) with Principal Component Analysis of the final scale identifying the factor loading of each item (Table 6). 

Internal consistency reliability was 0.906 for the final seven items, indicating an excellent degree of reliability. Reliability for the subscales ranged between 0.78 and 0.88. All reliability measures across population groups and subscales are summarised in Table 7. In addition to Cronbach’s alpha, an alternative measure of internal consistency was also calculated, and the mean inter-item correlations are summarised in Table 8. Temporal stability was calculated by the two-way, mixed, absolute measure Intraclass Correlation Coefficient (ICC) and received 0.970 (95% CI 0.957–0.979), *p* = 0.000. The ICC for the subscales was subscale 1 = 0.93, subscale 2 = 0.92, subscale 3 = 0.93 and subscale 4 = 0.96. The Standard Error of Measurement for the scale was 2.43 and the Minimal Detectable Change (MDC) was 6.7 with a 95% confidence interval. The MDC will be interpreted as the minimum amount of change in score that is clinically significant for respondents across 1–3 weeks.

## 4. Discussion

This paper presents a new ADE screening tool with both clinical and research applications for DE screening in current and former athletes. The ADE screening tool has been developed thoroughly through a comprehensive methodology utilising both CTT and IRT to confirm a rigorous level of validation and reliability in two diverse athletic population groups. This is the first scale to be developed for former athletes, the first screening tool to measure risk of DE in adult athlete populations groups and the first scale to be developed in the ED field with IRT methodologies. Future implementation of the ADE screening tool will enable timely and fast screening for DE with the aim of preventing the development of EDs in diverse athletic populations across sports and sporting competition levels.

This ADE screening tool developed in the current study is particularly unique for its varied and rigorous features. The scale enables categorisation of individuals’ scores within a range of DE indications from minimal or no indication of DE (total score < 25), moderate indication (total score between 25 and 32), high indication of DE (total score between 33 and 44) and very high indication of DE (total score ≥ 45). The scale is made up of four unidimensional subscales that represent distinct aspects of the DE conceptualisation. These subscales can be utilised to identify individuals with specific presentations relating to (1) food and energy control, (2) bingeing, (3) body control and (4) body discontent. Cronbach’s alpha of the scale was 0.91 and ranged between 0.78 and 0.88 for the four subscales. The intentionally, highly sensitive cut-off point for a moderate indication of DE was a total score of 25 (sensitivity 99.3, specificity 27.3%) to ensure that all current and former athletes across varying presentations were captured for risk. The scale is suitable to detect clinically significant changes in DE indication with a score change of seven or greater. Utilising cut-off scores ≥ 33, 59.9% of current and former athletes in the development sample (*n* = 851) had scores suggesting a high indication of DE and 28.1% with scores suggesting a very high indication of DE. The ADE can be utilised by coaching and medical staff to screen athletes for DE, which could be performed systematically, e.g., in pre-season/start of season or as needed if coach identifies concerns. It has been developed to be used by/for athletes at any level and in any sporting code. If an athlete’s score identifies them to be at moderate, high or very high risk of DE, they should be referred to a medical doctor who can then determine what further interventions/referrals are needed.

The ADE screening tool challenges previous constructs of EDs and DE upon which previous scales have been developed from. The EAT-26 was developed primarily for cis-female presentations with items explicitly relating to vomiting and desires for an empty stomach with significant limitations for cis-males, let alone gender diverse individuals [9,22]. Robust scales such as the EDE-Q also have limited application in athletes due to the specific wording of certain items, which may cause athletes to not relate to items because of the cultural attitudes of their sporting environment [1]. For example, eating an unusually large amount compared to others may be a normal and essential behaviour for athletes with higher energy demands and not a sign of an ED. Newer athlete DE- and ED-specific scales such as the DESA-6 [8] and EDSA [15] are excellent examples of quick, easy-to-use screening tools for adolescents and adults, respectively. However, limitations of these tools are their ability to break down different components of DE that may affect athletes differently, e.g., bingeing as a primary distressing element of DE or DE cognitions without behaviours. The ADE screening tool offers merit in these instances as it provides greater clinical application and detailed information relating to the four components of DE (food and energy control, bingeing, body control and body discontent).

A strength of the scale development was the inclusion of over 800 participants in the development sample (*n* = 851). The population of this main sample was overwhelmingly from Australia (35.4%) and the USA (15.0%), with most participants identifying as white or Caucasian for their self-identified cultural background. This potentially provides unwanted homogeneity to the background of the participants as it is not representative of the broader population nor athletic populations. In addition, there was some homogeneity of the qualitative populations explored to expand upon the conceptualisation of DE in athletic population groups. Therefore, further research is needed recruiting across a broader range of social demographics, nations and non-leanness sports to further challenge and expand upon the conceptualisation of DE. 

It is evident that sporting research has a long way to go when exploring the complexity of gender as a social construct [48,49,50]. The main development part of this current study aimed to be inclusive outside of the sex binary (male and female) but were limited largely by the categories of sporting competitions. Our study included eight participants who did not preferentially identify as cis-male or cis-female. By having an inclusion criterion that did not exclude individuals outside of the sex binary, we were able to consider the merit of items if they were gender inclusive, i.e., that they did not relate specifically to one type of gendered body ideal or have any specific gendered language in them. Ideally, more individuals with differing gender experiences, particularly that of transgender, non-binary and intersex current and former athletes, would have been recruited to both validate and evaluate the ADE screening tool, however, were limited by the project scope.

## 5. Conclusions

The ADE screening tool is the first psychological scale to specifically measure risk of DE in adult athletes, differentiating it from EDs, and the first such tool to be validated in a broad range of current and former athletes. By reconceptualising DE for the development of this scale, we have enhanced the understanding of DE so that sports supporters, current athletes, former athletes, coaches and clinicians, specifically, dietitians, exercise physiologists, physicians and psychologists working in EDs and sport, have a greater awareness surrounding the nuanced presentations of DE in athletic populations. This tool will enable screening for DE with the aim of preventing the development of EDs in the challenging sporting cultures where DE is often normalised and at times framed as being essential to performance.

## Figures and Tables

**Table 1:** Quantitative participant recruitment purpose and relevant statistical analyses.

Participant Group and Purpose	Recruitment Method	Number of Participants (*n*)	Relevant Statistical Analysis
1. Expert Validation—Selected global experts in the field of sports nutrition and/or eating disorders	Purposive sampling via email invitation	11	Content Validation Qualitative Feedback
2. Pilot Study of Current and Former Athletes	Purposive sampling via social media	51	Content Validation Qualitative Feedback
3. Development Sample of Current and Former Athletes	Convenience sampling via social media (including the use of paid advertising) and colleague email recruitment	851	Initial Item Evaluation Exploratory Factor Analysis Confirmatory Factor Analysis Rasch Analysis Cross-Cultural Validity Construct Validity Internal Consistency Reliability
4. Test-Retest Sample of Current and Former Athletes	Purposive sampling for follow up from development sample	125	Test-Retest Reliability

**Table 2:** Additional demographic information for the development sample.

Education	Tertiary Qualification	64.9% (*n* = 552)		
Employment	Full-time employment	33.3% (*n* = 283)		
Nationality	Australia	35.4% (*n* = 301)	Greece	4.5% (*n* = 38)
	USA	15.0% (*n* = 128)	Ireland	4.5% (*n* = 38)
	Singapore	8.8% (*n* = 75)	New Zealand	3.5% (*n* = 30)
	South Africa	6.8% (*n* = 58)	Hungary	2.0% (*n* = 17)
	UK	6.2% (*n* = 53)	Russia	1.2% (*n* = 10)
	Canada	5.3% (*n* = 45)	Other	6.8% (*n* = 58) *
Sporting Categories	Ball Sports	31.6% (*n* = 169)	Weight Class	7.1% (*n* = 60)
	Endurance	28.1% (*n* = 239)	Technical	4.5% (*n* = 38)
	Power	11.5% (*n* = 98)	Antigravitational	1.3% (*n* = 11)
	Aesthetic	7.8% (*n* = 66)	Other	8.2% (*n* = 70)
Highest Competition Level	Club	22.2% (*n* = 189)	National	35.7% (*n* = 304)
	State	16.9% (*n* = 144)	International	25.1% (*n* = 214)
Previous Eating Disorder (23.6%, *n* = 201)	Anorexia Nervosa	8.1% (*n* = 69)	Binge Eating Disorder	3.1% (*n* = 26)
	Bulimia Nervosa	4.2% (*n* = 36)	OSFED	2.1% (*n* = 18)
	Orthorexia Nervosa	2.5% (*n* = 21)	UFED	1.6% (*n* = 14)
	Other	2.0% (*n* = 17)		
Current Eating Disorder	No	90.1% (*n* = 773)	Yes	9.2% (*n* = 78) **

* Other nationalities included Argentina (*n* = 3), Belgium (*n* = 1), Chile (*n* = 1), Colombia (*n* = 1), Czech Republic (*n* = 1), Denmark (*n* = 6), Dominican Republic (*n* = 1), France (*n* = 4), Germany (*n* = 5), Hong Kong (*n* = 1), India (*n* = 1), Indonesia (*n* = 1), Israel (*n* = 1), Italy (*n* = 5), Japan (*n* = 1), Kazakhstan (*n* = 1), Lebanon (*n* = 1), Lithuania (*n* = 1), Malaysia (*n* = 2), Mexico (*n* = 2), Netherlands (*n* = 2), Norway (*n* = 1), Philippines (*n* = 2), Poland (*n* = 3), Slovakia (*n* = 1), South Korea (*n* = 1), Spain (*n* = 3), Switzerland (*n* = 1), Turkey (*n* = 1), Ukraine (*n* = 1), Venezuela (*n* = 1) and Zambia (*n* = 1). ** Females with current ED (*n* = 71), males with current ED (*n* = 6), non-binary individuals with a current ED (*n* = 1), current athletes with a current ED (*n* = 40) and former athletes with a current ED (*n* = 38).

**Table 3:** Summary of overall Rasch model fit statistics for all four factors across four population groups.

	Factor 1 (*n* = 5 Items)	Factor 2 (*n* = 3 Items)	Factor 3 (*n* = 4 Items) **	Factor 4 (*n* = 5 Items)	Whole Scale (*n* = 17 Items)
Item Fit Residual (mean ± Standard Deviation (SD)	1.03 ± 0.43	0.52 ± 1.64	0.65 ± 1.40	0.27 ± 1.80	3.38 ± 0.43
Person Fit Residual (mean ± Standard Deviation (SD)	1.05 ± 0.32	−0.47 ± 1.06	−0.53 ± 1.38	−0.38 ± 1.10	1.58 ± 0.34
Item Chi Square Statistic	57.8	30.7	41.7	50.2	495.2
Degrees of Freedom	45.0	27.0	45.0	45.0	153.0
Chi Square Probability *	0.095, *p* > 0.010	0.284, *p* > 0.017	0.610, *p* > 0.012	0.280, *p* > 0.010	0.000, *p* < 0.003
Person Separation Index	0.74	0.75	0.75	0.84	0.90
Coefficient Alpha	0.78	0.82	0.80	0.88	0.91

* Adjusted for Bonferroni correction, where 0.05 is divided by the number of items analysed. ** Differential Item Functioning (DIF) split for athletic status on item 14; Cronbach’s alpha not with DIF split.

**Table 4:** Determined specificity and sensitivity of cut-off points for the ADE screening tool.

		ADE Screening Tool	Subscale 1 Food Control	Subscale 2 Binge	Subscale 3 Body Control	Subscale 4 Body Discontent
Moderate Disordered Eating Indication	Cut-Off	25.0				
	Sensitivity	99.3%				
	Specificity	27.3%				
High Disordered Eating Indication	Cut-Off	33.0	10.5	6.5	11.5	14.5
	Sensitivity	96.8%	85.6%	61.2%	65.5%	87.8%
	Specificity	49.7%	78.3%	64.2%	79.9%	75.9%
Very High Disordered Eating Indication	Cut-Off	45.0				
	Sensitivity	79.9%				
	Specificity	84.7%				

**Table 5:** Component Correlation Matrix for *n* = 17 items.

	Factor 1	Factor 2	Factor 3	Factor 4
Factor 1	1			
Factor 2	0.444	1		
Factor 3	0.319	0.206	1	
Factor 4	0.418	0.196	0.345	1

**Table 6:** Exploratory factor analysis—Principal Component Analysis of final ADE screening tool (*n* = 17 items).

Item	Pattern Coefficients				Structure Coefficients				Communalities
	Factor 1	Factor 2	Factor 3	Factor 4	Factor 1	Factor 2	Factor 3	Factor 4	
I feel bad when an athlete has a better-looking body than mine	0.841				0.853				0.728
I am dissatisfied with my body size or shape	0.853				0.837				0.707
I compare my body to other athletes or my former self	0.778				0.826				0.711
My performance or mood is influenced by how I feel about my body	0.692				0.792				0.651
I fear fat gain or muscle loss	0.647				0.763				0.642
Once I start eating, I find it hard to stop		0.863				0.874			0.774
There are certain foods I can not control myself around		0.867				0.859			0.748
I overeat when I am allowed to eat freely, i.e., off-season or a buffet		0.853				0.834			0.721
I am motivated to train harder to influence my body shape or weight			0.849				0.863		0.748
I will perform extra exercise to influence my body shape			0.826				0.855		0.746
I look to control my food when I want more out of my body			0.524				0.650		0.550
To change my body, I cut back on foods or ingredients			0.383				0.587		0.569
I carefully plan and think about what I eat				0.830				0.785	0.658
I avoid social situations if there will be foods I do not feel comfortable eating				0.621				0.705	0.561
I find spontaneous eating decisions challenging				0.584				0.681	0.583
If I have not exercised that day I will limit my food				0.527				0.680	0.568
I think about the calories/kilojoules I am burning when I train or exercise				0.364				0.580	0.503

Factor 1 = subscale 1: food and energy control; Factor 2 = subscale 2: bingeing, Factor 3 = subscale 3: body control; and Factor 4 = subscale 4: body discontent.

**Table 7:** Internal consistency reliability (Cronbach’s alpha) for subscales and the four population groups.

	ADE Screening Tool	Subscale 1 Food Control	Subscale 2 Binge	Subscale 3 Body Control	Subscale 4 Body Discontent
Current Males	0.875	0.652	0.766	0.791	0.858
Former Males	0.873	0.640	0.815	0.693	0.836
Current Females	0.918	0.810	0.840	0.832	0.902
Former Females	0.931	0.846	0.849	0.829	0.896

**Table 8:** Mean inter-item correlations for the ADE scale and subscales across population groups.

	ADE Screening Tool	Subscale 1 Food Control	Subscale 2 Binge	Subscale 3 Body Control	Subscale 4 Body Discontent
Current Males	0.289	0.272	0.522	0.487	0.546
Former Males	0.287	0.262	0.595	0.361	0.504
Current Females	0.402	0.460	0.637	0.554	0.649
Former Females	0.523	0.523	0.651	0.548	0.632

## Data Availability

The raw data supporting the conclusions of this article will be made available by the authors on request.

## Appendix A

Recommended participant numbers for validation and reliability analyses as per the COSMIN Study Design Checklist [27].

**Statistical Analysis**	**Participant Number (*n*)**
Construct Validation (Discriminative or Known Groups)	≥100
Construct Validation (Hypothesis Testing)	≥100
Content Validation (Qualitative)	≥7
Content Validation (Quantitative)	≥50
Criterion Validation	≥50 per group
Cross-Cultural Validation (Regression Analysis)	≥200
Cross-Cultural Validation (Rasch Analysis)	≥200
Cross-Cultural Validation (Multi-Group Confirmatory Factor Analysis)	≥100 per group Or 7× the number of items
Factor Analysis	≥100 Or 7× the number of items
Factor Analysis (Rasch Analysis)	≥200
Internal Consistency	≥100
Measurement Error	≥100
Test-Retest Reliability	≥100
